# Monodisperse Porous Silica/Polymer Nanocomposite Microspheres with Tunable Silica Loading, Morphology and Porosity

**DOI:** 10.3390/ijms232314977

**Published:** 2022-11-29

**Authors:** Julia C. Steinbach, Fabio Fait, Hermann A. Mayer, Andreas Kandelbauer

**Affiliations:** 1 Process Analysis & Technology, Reutlingen Research Institute, Reutlingen University, Alteburgstraße 150, 72762 Reutlingen, Germany; 2 Institute of Inorganic Chemistry, University of Tübingen, Auf der Morgenstelle 18, 72076 Tübingen, Germany; 3 Institute of Wood Technology and Renewable Materials, Department of Material Sciences and Process Engineering (MAP), University of Natural Resources and Life Sciences, Gregor-Mendel-Straße 33, 1180 Vienna, Austria

**Keywords:** nanocomposites, porous microspheres, design of experiment, response surface methodology, rational design, sol-gel processing

## Abstract

Hybrid organic/inorganic nanocomposites combine the distinct properties of the organic polymer and the inorganic filler, resulting in overall improved system properties. Monodisperse porous hybrid beads consisting of tetraethylene pentamine functionalized *poly*(glycidyl methacrylate-*co*-ethylene glycol dimethacrylate) particles and silica nanoparticles (SNPs) were synthesized under Stoeber sol-gel process conditions. A wide range of hybrid organic/silica nanocomposite materials with different material properties was generated. The effects of n(H_2_O)/n(TEOS) and c(NH_3_) on the hybrid bead properties particle size, SiO_2_ content, median pore size, specific surface area, pore volume and size of the SNPs were studied. Quantitative models with a high robustness and predictive power were established using a statistical and systematic approach based on response surface methodology. It was shown that the material properties depend in a complex way on the process factor settings and exhibit non-linear behaviors as well as partly synergistic interactions between the process factors. Thus, the silica content, median pore size, specific surface area, pore volume and size of the SNPs are non-linearly dependent on the water-to-precursor ratio. This is attributed to the effect of the water-to-precursor ratio on the hydrolysis and condensation rates of TEOS. A possible mechanism of SNP incorporation into the porous polymer network is discussed.

## 1. Introduction

The combination of organic and inorganic materials provides composites exhibiting different properties than the inorganic and organic components alone and is, therefore, of great interest in material sciences [1,2,3,4]. The incorporation of inorganic components, such as silica nanoparticles (SNPs), into organic networks allows for the design of material properties which affect their thermal and/or mechanical stability [5,6] and electronic behavior [7] and enhance biocompatibility [8]. Thus, hybrid materials are applied in catalysis [9] and separation technologies [10] and as drug delivery systems [8,11]. Moreover the organic component can function as a sacrificial template to generate defined porous inorganic structures [12,13,14,15].

For many applications, defined specific surface areas are favorable, which are achieved by introducing porous networks into the material. A good effective accessibility of the porous material for diffusion controlled processes, such as separation processes or catalysis, is achieved by mesopores and/or macropores [16]. The properties of porous *poly*(glycidyl methacrylate-*co*-ethylene glycol dimethacrylate) (*p*(GMA-*co*-EDMA)) particles in the micron scale are specifically adjustable [17,18,19,20], which makes them an adaptable polymer matrix with a broad pore size distribution.

Nanocomposites are multiphase materials with at least one component in the nanometer range [21]. The preparation of hybrid organic/silica nanocomposites can be achieved by (1) the blending of dispersed polymer and silica nanoparticles, (2) the in situ polymerization of monomers in the presence of silica and (3) performing the sol-gel process from precursors in the presence of a polymer matrix [22]. The sol-gel process allows for the preparation of tailored silica materials with, e.g., defined nanoparticle sizes and dispersion under mild process conditions and is therefore particularly suitable for preparing tailored organic/silica composites [23].

Discrete SNPs with narrow size distributions are readily synthesized under basic reaction conditions by the Stoeber method. The sizes of silica nanoparticles obtained with this method vary between 0.05 µm and 2.0 µm [24]. The Stoeber method exploits the basic hydrolysis and condensation reactions of alkoxysilanes in alcoholic media [24]. The hydrolysis of the precursor (Equation (1)), e.g., tetraethyl orthosilicate (TEOS), proceeds under the formation of silanol groups (n = 1–4). The condensation with the formation of siloxane bonds and entire silica networks occurs either between silanol groups as water condensation (Equation (2)) or between silanol and ethoxy groups as alcohol condensation (Equation (3)).

Hydrolysis:  Si(OC_2_H_5_)_4_ + n H_2_O ⇌ Si(OC_2_H_5_)_4-n_(OH)_n_ + n C_2_H_5_OH (1)

Water condensation:  ≡Si(OH) + (HO)Si≡ ⇌ ≡Si–O–Si≡ + H_2_O (2)

Alcohol condensation:  ≡Si(OC_2_H_5_) + (HO)Si≡ ⇌ ≡Si–O–Si≡ + C_2_H_5_OH (3)

Immediately after the addition of TEOS to an ammonia solution, progressing hydrolysis and condensation of the precursor starts. After reaching a critical supersaturation concentration, neighboring silanol monomers condense under the formation of clusters, which collapse to form primary particles [25,26,27]. There are two prevailing models to describe the further growth mechanism of silica particles: the monomer addition model [28], which depicts the growth as the addition of the monomer to already-formed primary particles, and the aggregation-only model [29], which explains particle growth through the aggregation of primary particles and the subsequent addition of oligomers. Small silica particles are not stable and aggregate, while stable large silica particles grow by the addition of monomers [30]. The size of the resulting SNPs depends on the relative rates of the hydrolysis and condensation reactions of the precursor. These are controlled by the relative concentrations of ammonia, TEOS and water as well as the reaction temperature and the alcohol chosen as the solvent [31].

As the sol-gel process is very susceptible to changes in the process conditions, a univariate study focusing on the effects of single factors only cannot depict the system complexity. The one-factor-at-a-time approach does not allow for the covering of a multidimensional experimental space and therefore for the identification of the synergistic interplay between two factors. The observation of the influence of single-factor variations cannot straightforwardly be transferred to other systems settings, since even small changes have a strong impact on the results. This is reflected by the sometimes even contradicting conclusions when the experimental findings of different studies are compared with one another, although they are self-consistent by themselves. Especially for the sol-gel process, where the relative rates of hydrolysis and condensation are affected by many parameters [25,26,30], a study where at least two process factors are simultaneously varied is required to understand the impacts and possible interaction effects between experimental variables. Response surface methodology (RSM) provides a statistical and systematic approach for the observation and quantification of not only linear but also non-linear behavior. Moreover, possible synergistic interactions between process factors are identified and provide detailed causal models for multiple process factors [17,32,33,34]. This approach has been used to study a wide range of chemical processes [35,36,37,38] and has also been successfully applied for the preparation of SNPs [39,40,41,42,43].

In this study, micron-sized monodisperse porous hybrid organic/silica hybrid beads (HBs) are generated by performing a sol-gel process under Stoeber reaction conditions in the presence of porous amino-functionalized *p*(GMA-*co*-EDMA) particles (p@TEPA) as hard templates. The hybrid beads consist of two interpenetrating networks: the organic network of the template, which remains unchanged due to the mild reaction conditions, and the new silica network, which is composed of aggregated silica nanoparticles of different sizes [12,14].

The aim is to achieve a range of such hybrid materials with different and defined properties such as SiO_2_ content, median pore size, specific surface area and pore volume. Thus, the two critical process factors for the sol-gel process, the water-to-precursor ratio (n(H_2_O)/n(TEOS)) and the ammonia concentration (c(NH_3_)) were investigated in the presence of a porous polymeric template. These were systematically changed according to a face-centered-central composite design (FCD) to establish response surface models describing the process factor effects on the morphological characteristics. Moreover, a possible mechanism of silica nanoparticle incorporation into the porous polymer matrix is proposed and discussed.

## 2. Results

**Figure 1:**
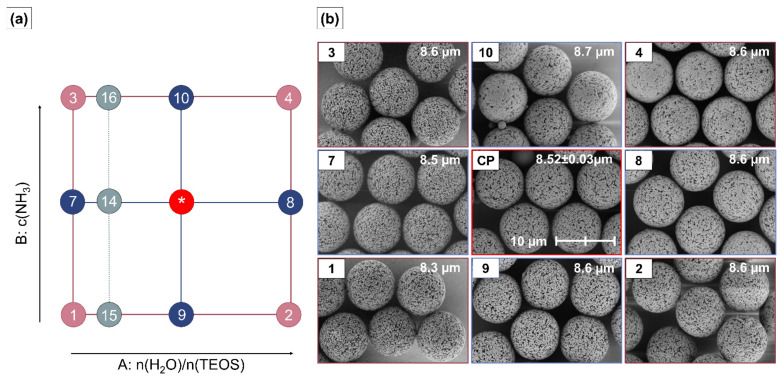
(**a**) Illustration of the face-centered-central composite design including the additional points HB14–16 (**b**) Systematic overview over SEM images at 5000× magnification of HBs. The median size of each sample is indicated in the respective image corner. For the CPs, the average particle size and its standard deviation over all five samples is given. The SEM image of HB13 is shown as a representative example for the CPs. Factorial design points (HB1–4) are displayed in dark red, center points (CPs) are displayed in red (HB5–6, HB11–13), axial design points (HB7–10) are displayed in blue and additional design points are shown in grey (HB14–16). SEM images of the additional design points HB14–16 are shown separately in Appendix B Figure A1. * indicates the center point experiments at intermediate factor level settings.

The preparation of micron-sized porous hybrid beads (HBs) with a tunable ratio of organic components to silica and morphological properties was systematically investigated using response surface methodology (RSM) (Figure 1). The organic porous *p*(GMA-*co*-EDMA) template was prepared by seeded swelling polymerization (see Appendix A) [17]. Tetra ethylene pentamine (TEPA) was covalently bound to the porous polymer surface via ring opening reactions of the prevalent epoxy groups [44,45]. The TEPA functionalization of polymer particles (p@TEPA) has been shown to provide a well-suited surface modification for depositing silica into the polymer network [12,46]. The prepared p@TEPA template had a median particle size of 8.3 µm and was highly monodisperse, with a d_90_/d_10_ value of 1.04. The median pore diameter (Φ_50_) of the p@TEPA was 13.0 nm in combination with a specific surface area (SSA) of 63.79 m²∙g^−1^ and a pore volume (V_p_) of 0.12 cm^3^∙g^−1^.

The hybrid beads HB1-HB16 consisting of p@TEPA/SiO_2_ were prepared by varying the sol-gel process conditions according to Table 1 in the presence of the p@TEPA template. With RSM, linear and non-linear effects of the process factors n(H_2_O)/n(TEOS) and c(NH_3_), as well as their possible synergistic interactions, on the material properties SiO_2_ content, Φ_50_, V_p_, SSA and size of the SNPs were analyzed using a face-centered-central composite design (FCD). Figure 1a schematically shows the experimental space covered by the FCD. Selected HBs which result from different process factor settings according to the RSM are shown in Figure 1.

Table 1 summarizes the factor-level settings of n(H_2_O)/n(TEOS) and c(NH_3_) and the resulting values of the measured response properties. The samples are listed in the Yates standard order. In addition to the HB samples, the material properties of the template (p@TEPA) are given for comparison. One of the axial design points, HB9, was identified as an outlier based on the extremely high values of its residuals for the pore volume and silica content. Thus, it was not used for model building. Model quality likewise improved for the models of all other targeted responses as well upon the omission of HB9.

### 2.1. Size and Dispersity

The particle sizes of the produced HB1–HB16 vary in a narrow range from 8.3 to 8.6 µm (8.5 ± 0.1 µm) and are monodisperse, with a particle size distribution with d_90_/d_10_ values between 1.05 and 1.15 (1.08 ± 0.08). SEM images of the particles obtained at each design point are given in Figure 1b. Thus, the polymeric hard template is mainly determining the size of the HBs within the examined design space, and the narrow dispersity of the template particles is very well reproduced by the HBs. However, a small positive effect of the ratio n(H_2_O)/n(TEOS) on the particle size was found to be statistically significant (see Table 2). This indicates that an increase in n(H_2_O)/n(TEOS) leads to a minor increase in the HB particle size. The rather poor values for the model quality parameters R² = 0.3642 and R²_pred_ = 0.1684 indicate that an effect can be detected; however, it is too weak to be quantitatively modeled sufficiently well within the range of the examined design space.

### 2.2. SiO_2_ Content

The amount of silica, which is deposited in the pores of the template, was determined thermogravimetrically (see Section 4.5). The SiO_2_ content in the hybrid materials is described by a statistically highly significant model (*p* < 0.0001) with a high robustness R²_pred_ = 0.9459 and, hence, a good predictive power (Table 2).

The model effect terms A–n(H_2_O)/n(TEOS) and A², as well as B–c(NH_3_), are statistically highly significant at an α-value of 0.05. The effect strengths in terms of coded factors are given in Equation (4):

SiO_2_ = 25.49 + 13.34 A + 3.55 B − 11.80 A² (4)

**Figure 2:**
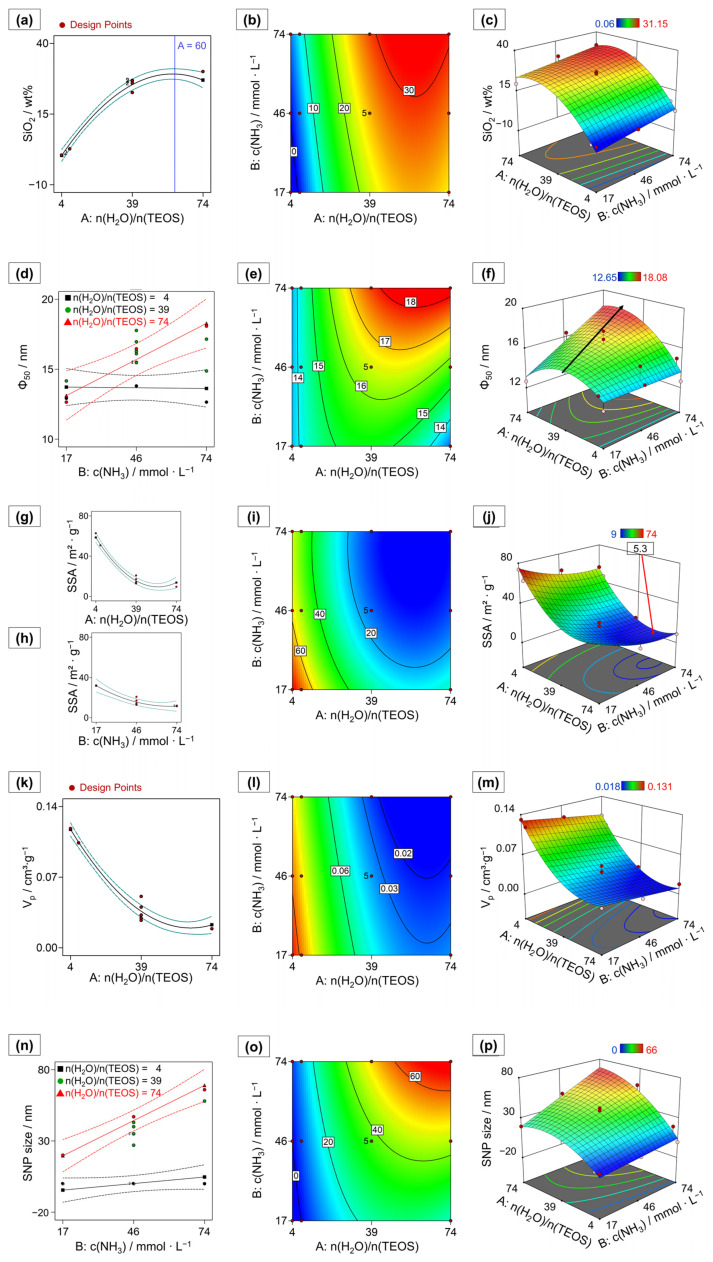
Overview over all relevant model graphs–one factor effect plots, depicting the effect of a single factor on the response at a medium level of the other studied process factor (**a**,**g**,**h**,**k**); interaction plots showing the dependence of the response on the settings of the two synergistically interacting factors (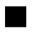 = low setting, 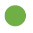 = medium setting, 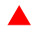 = high setting) and visualizing the strengths of the synergistic interactions (**d**,**n**); contour plots displaying a heatmap for each response variable dependent on both process factors (**b**,**e**,**i**,**l**,**o**); response surface plots showing the values for the responses predicted by the response surface model across the design space for each combination of process factor settings (**c**,**f**,**j**,**m**,**p**). Note that, in the 3D-plots (**j**,**m**), the axes of n(H_2_O)/n(TEOS) are given in opposite direction to (**c**,**f**,**p**) for better visibility. Dashed lines indicate 95% confidence intervals for one factor and interaction plots. Red areas correspond to high and blue areas correspond to low response values in the response surface models. The responses are as follows: (**a**–**c**) SiO_2_ content, blue line indicates maximum SiO_2_ deposition; (**d**–**f**) median pore size (ϕ_50_), the black arrow indicates a shift in maximal ϕ_50_ across the design space; (**g**–**j**) specific surface area (SSA) with the minimal SSA within the experimental space indicated by a flag; (**k**–**m**) pore volume (V_p_); (**n**–**p**) SNP size.

The stoichiometric water-to-precursor ratio shows by far the strongest effect and a non-linear behavior. The positive effect leads to an increase in the amount of deposited silica in the polymer with an increasing molar ratio. The negative A² effect term describes the non-linear behavior of the increase in silica deposition with an increasing n(H_2_O)/n(TEOS) ratio. At a stoichiometric water-to-precursor ratio of ~60:1, the silica deposition reaches a maximum (Figure 2a–c).

### 2.3. Pore Size

**Figure 3:**
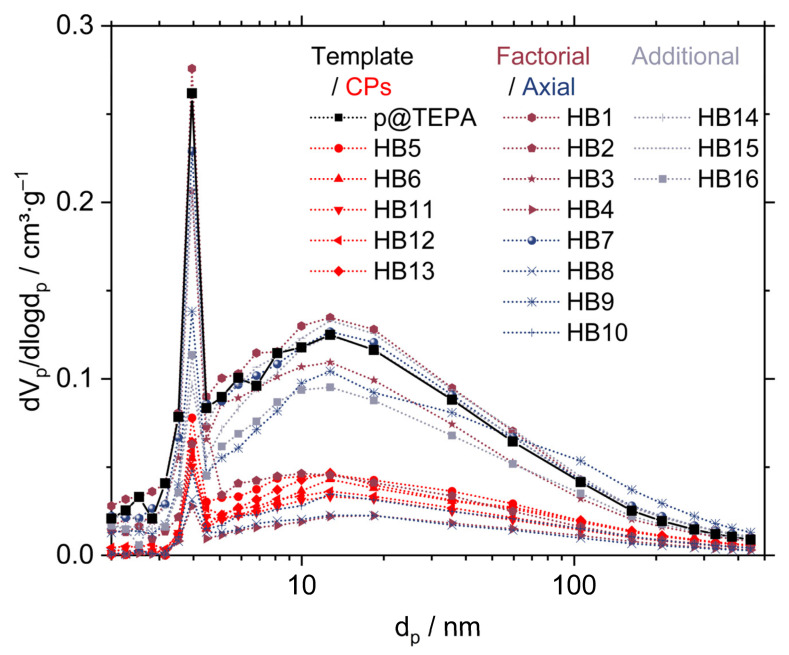
Pore size distribution of the porous p@TEPA template (solid line) and porous HBs (dotted lines). The template is shown in black, factorial design points are shown in dark red, center points (CPs) are shown in red, axial design points are shown in blue and additional design points are shown in grey.

Pore characteristics were determined by nitrogen adsorption measurements at 77 K. The pore size was calculated according to the Barrett–Joyner–Halenda (BJH) method from the desorption curve. For response surface modeling, the median of the pore size was used. The pore size distributions of all HBs, after the incorporation of in situ-prepared SNPs into the porous template network, and the template are shown in Figure 3, and the median pore size values are listed in Table 1.

The pore size distribution of the HBs is mainly determined by the pore size distribution already present in the p@TEPA template. It displays a very wide distribution from the meso- to macroporous range. Compared to the template, the median of the pore sizes is shifted to larger pores in the hybrid materials (see Table 1).

With a correlation coefficient of R² = 0.8299 and a regression coefficient of prediction of R²_pred_ = 0.5529, the model (*p* < 0.001) can describe the pore size median well. The median of the pore sizes is statistically significantly affected by the effect terms A–n(H_2_O)/n(TEOS) and B–c(NH_3_), the synergistic interaction effect term AB between the water-to-precursor ratio and ammonia concentration and the non-linear effect term A². The relative importance of the factors is evident from Equation (5), the model equation in terms of coded factors:

Φ_50_ = 16.49 + 1.01 A + 1.26 B + 1.31 AB − 1.80 A² (5)

The synergistic interaction effect term AB quantifies the interdependence between the two factors. Consequently, restricting the discussion to only one of the factors is not meaningful, since the behavior of one factor depends on the factor-level setting of the other and changes while dependent on the second factor involved in the interaction. Figure 2d shows the two-factor interaction (2FIA) plot for the median pore size, with the ammonia concentration depicted on the abscissa. For small ratios A–n(H_2_O)/n(TEOS), the ammonia concentration has very little or no effect on the median pore size, while for large n(H_2_O)/n(TEOS) ratios, the median pores size distinctly increases with an increasing B–c(NH_3_). Increasing the ratio n(H_2_O)/n(TEOS) amplifies the effect of the NH_3_ concentration. Comparing HB1 (−/−) and HB3 (−/+), the median pore size is almost constant (12.9 nm and 12.6 nm, respectively), while for a high water-to-precursor ratio, the median pore size increases strongly from HB2 (+/−) at 12.7 nm to HB4 (+/+) at 18.1 nm with an increasing ammonia concentration.

The non-linear A^2^ term shows that the increase in the median pore size towards higher values with increasing n(H_2_O)/n(TEOS) becomes less pronounced at higher levels of n(H_2_O)/n(TEOS). It also indicates that there is a maximum mean pore size, the location of which depends on the corresponding level of ammonia concentration used. This is especially visible in the trajectory of the contour plot (Figure 2e) and in the curvature of the response surface (Figure 2f) at high ammonia concentrations. The shift in the maximum pore size median is indicated by an arrow.

The combination of the non-linear A^2^ term with the synergistic interaction term AB results in a twisted response surface with a steep ascent and a shifting maximum which shows a high sensitivity towards small changes in the factor-level settings of both the n(H_2_O)/n(TEOS) ratio and the ammonia concentration. This sensitivity is also reflected by the relatively large statistical variance of the pore sizes observed within the replicated CP experiments.

### 2.4. Specific Surface Area

For many applications, the specific surface area (SSA) is of great importance, since it is crucial for the surface available for, e.g., the amount of functionalization or adsorption. The SSA was determined by nitrogen adsorption measurements under the application of the Brunauer–Emmet–Teller (BET) theory [47]. All samples showed values for the C parameter from the BET equation, referring to the surface interaction energy, between 48.7 (HB3) and 133.7 (HB2). This indicates a sufficiently well discriminated knee in the adsorption isotherm to determine the theoretical formation of the molecular monolayer [47]. The SSAs of the prepared HBs are modeled with a high predictive power and robustness (R² = 0.9840 and R²_pred_ = 0.9535, respectively). The model is highly statistically significant (*p* < 0.0001) and includes the statistically significant factor effect terms A–n(H_2_O)/n(TEOS) and B–c(NH_3_), as well as both non-linear factor effect terms A² and B². No synergistic interactions between the two factors were found. The coded equation describing the relative effect strengths is given in Equation (6):

SSA = 15.59 − 22.38 A − 10.17 B + 20.57 A² + 6.22 B²(6)

The SSAs of the HBs are mainly determined by the ratio n(H_2_O)/n(TEOS), which has a strong negative effect. The SSAs decrease with an increasing water ratio. This effect is attenuated by the positive A² effect term accounting for the minimum which is observed at high n(H_2_O)/n(TEOS) ratios (Figure 2g).

A similar behavior is seen for increasing ammonia concentrations. The negative linear effect term B combined with the positive non-linear effect term B² indicate a decrease in the SSAs with increasing ammonia concentrations, which, again, levels off at higher concentrations (Figure 2h,i). A minimum in SSA of approximately 5.3 m²∙g^−1^ is found in the experimental space at A = 58 and B = 68 mmol∙L^−1^, which is highlighted in the response surface plot of Figure 2j.

### 2.5. Pore Volume

The pore volumes V_p_ of the HB samples which were determined at *p*/*p*_0_ = 0.95 are expressed by a highly statistically significant RSM (*p* < 0.0001) as a function of the process factor effect terms A–n(H_2_O)/n(TEOS) and B–c(NH)_3_ and the non-linear factor effect term A². The dependence of V_p_ on the process factors is given in terms of coded factor effect terms in Equation (7):

V_p_ = 0.0362 − 0.0473 A − 0.0122 B + 0.0388 A² (7)

The factor effect term A has the strongest influence determining the pore volume. The pore volume of the HBs decreases with an increasing water-to-precursor ratio. However, this decrease is attenuated at higher ratios by the positive nonlinear A² term (see Figure 2k).

In addition, increasing ammonia concentrations also lead to a decrease in pore volume, but this effect is much less pronounced and is only about one-fourth of the magnitude of the effect of factor A. This results in the highest pore volumes found at low factor-level settings for both factors A and B (Figure 2l,m). The model quality parameters R² = 0.9800 and R²_pred_ = 0.9635 indicate an excellent predictive power and model robustness.

### 2.6. Morphology and Silica Nanoparticle Size

**Figure 4:**
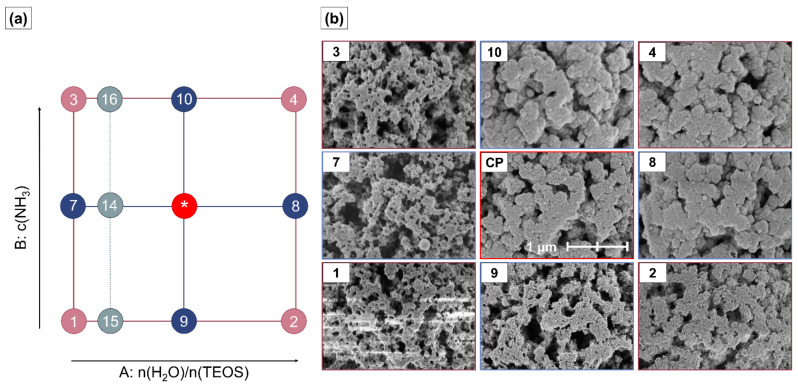
(**a**) Schematic representation of the design space covered by the factor-level combinations used in the face-centered-central composite design including the additional design points HB14–16. (**b**) Systematic overview over SEM images at 50,000× magnification of each sample obtained at the various factor-level combinations. A representative example of the center point (CP) combinations, HB13, is shown. Factorial design points (HB1–4) are displayed in dark red, center points are displayed in red (HB5–6, HB11–13), axial design points (HB7–10) are displayed in blue, and additional design points are shown in grey (HB14–16). Numbers reflect the Yates standard order. SEM images of the additional design points HB14–16 are shown separately in Appendix B Figure A2. * indicates the center point experiments at intermediate factor level settings.

**Figure A1:**
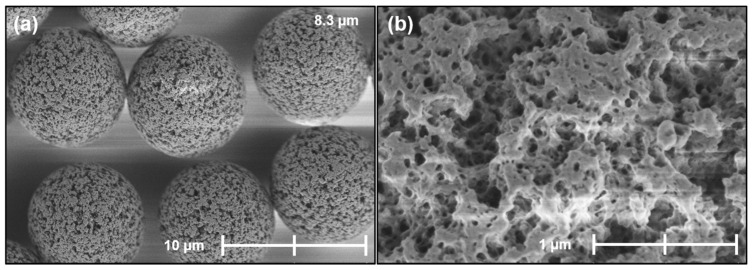
SEM image of *p*(GMA-*co*-EDMA) template batch at (**a**) 5000× and (**b**) 50,000× magnification. Particle size: 8.3 µm, d_90_/d_10_: 1.04, SSA: 63.79 m²∙g^−1^, V_p_: 0.12 cm³∙g^−1^, ϕ_50_: 14.47 nm.

**Figure A2:**
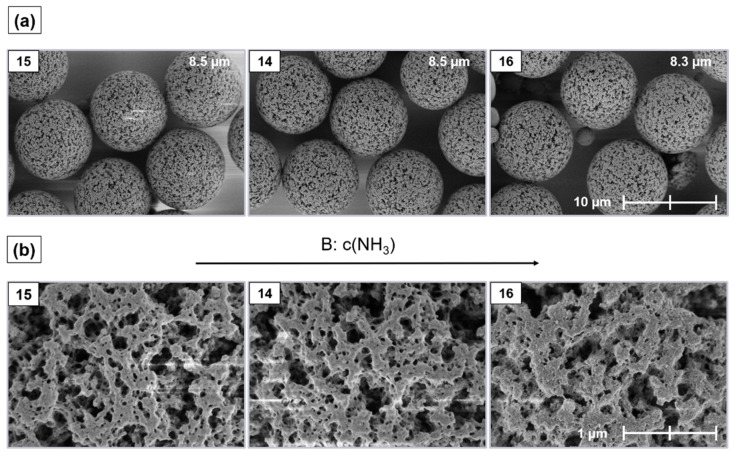
SEM image of the additional samples HB14–16 with n(H_2_O)/n(TEOS) = 8 sorted by increasing c(NH_3_) (l.t.r.) at (**a**) 5000× and (**b**) 50,000× magnification.

**Figure A3:**
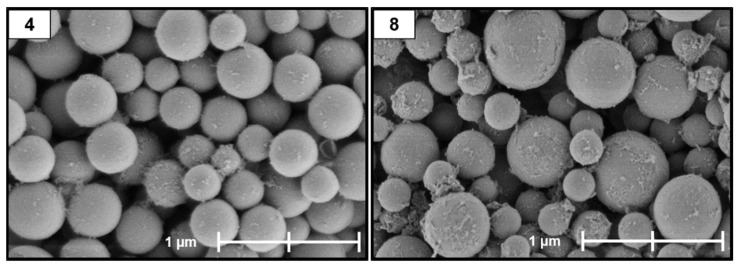
SEM image of filtrates with secondary particles of HB4 and HB8 at 50,000× magnification.

The morphology of the HBs is structured by the morphological properties of the template (Figure 4 and Figure A1). Textural changes result from the sol-gel process to varying degrees, dependent on the factor-level combinations. They are especially pronounced at high factor-level settings and are not visible below a certain threshold. Distinct differences in surface appearance are observed for the first time for the factor-level combinations of particle HB16 (n(H_2_O)/n(TEOS) = 8, c(NH3) = 74.1 mmol) (Appendix B Figure A2b). Instead of the smooth surfaces between the pore structures, small SNPs deposited in and onto the p@TEPA lead to an apparently rough surface texture. At medium and higher levels of the n(H_2_O)/n(TEOS) ratio, all produced HB particles exhibit a nanoparticulate surface texture (Figure 4). However, with higher ammonia concentrations, the discrimination between the SNPs from each other becomes less distinct. For HB4 and HB8, nonporous secondary particles were observed in the filtrate (Appendix B Figure A3). For HB4, these secondary particles were stable enough to be isolated. They showed a median size of ~300 nm and a d_90_/d_10_ value of 1.88. For HB8, agglomeration occurred during the evaporation of the solvent. Secondary particles were obtained with a median size of ~240 nm and a d_90/d10_ value of 2.05. However, since agglomeration occurred, this indicates that at least a portion of the secondary particles’ original size was below 50 nm [26].

The sizes of the SNPs are statistically significantly affected by the process factor A–n(H_2_O)/n(TEOS) in combination with the non-linear factor effect A², the linear effect B–c(NH_3_) and the synergistic interaction AB. The statistically significant model (*p* < 0.0001) shows a good data fit (R² = 0.9545) and allows for the prediction of new observations (R²_pred_ = 0.8874). The relative importance of the factor effects is again evident from the coefficients in Equation (8) in coded terms:

SNP size = 37.24 + 22.11 A + 14.65 B + 10.06 AB − 15.02 A²(8)

The positive direction of the synergistic interaction AB (Figure 2n) results in a pronounced increase in the SNP size that is dependent on increasing ammonia concentrations at the higher levels of n(H_2_O)/n(TEOS) ratios. The non-linear A² term is negative and reduces the increase in the size of the SNPs for higher n(H_2_O)/n(TEOS) levels, and the SNP size approaches a limiting value (at a factor-level setting of A ≈ 60).

## 3. Discussion

**Figure 5:**
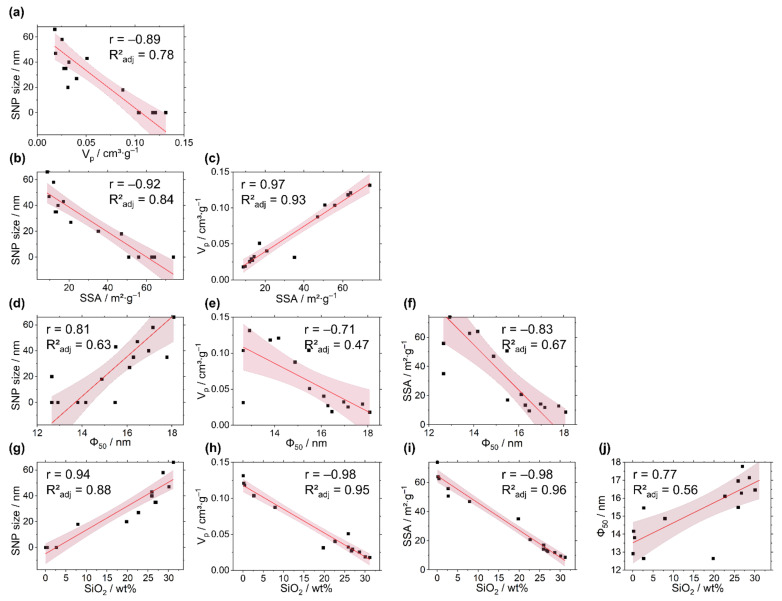
(**a**–**j**) Multiple scatterplots illustrating the correlations between response variables with Pearson correlation coefficients r and R²_adj_ fit parameters. Red lines indicate linear fits. 95% confidence intervals are given by shaded areas.

The response surfaces of the five response variables SiO_2_ content, ϕ_50_, SSA, V_p_ and the sizes of the SNPs show similarities with respect to the factor effect directions and indicate similar interdependencies (Figure 2). To illustrate how the various target responses depend on each other, all variables were plotted in pairs against each other in scatterplots. Figure 5 summarizes the resulting correlation matrix for each combination of response variables with Pearson r and R²_adj_ correlation coefficients. Pearson r is a measure of linear correlation with an indication of direct or indirect proportionality, and R²_adj_ is a measure for correlation adjusted by the number of model terms relative to the number of experiments to avoid overfitting.

All linear correlations are statistically significant at an α-level of 0.05. Here, the correlations with SiO_2_ content are generally the best (Figure 5g–j), while the median pore size shows a rather poor but still statistically significant correlation (Figure 5d–f). With an increase in SiO_2_ deposition, the V_p_ and SSA decrease, while the size of the SNPs at the HB surfaces increases (Figure 5g–i). Furthermore, the median of the pore size increases as well, which results in a decrease in the number of smaller pores. The positive correlation between the median pore size and SNP size shows that the median pore sizes of the HBs increase with the increasing size of SNPs (Figure 5d).

**Figure 6:**
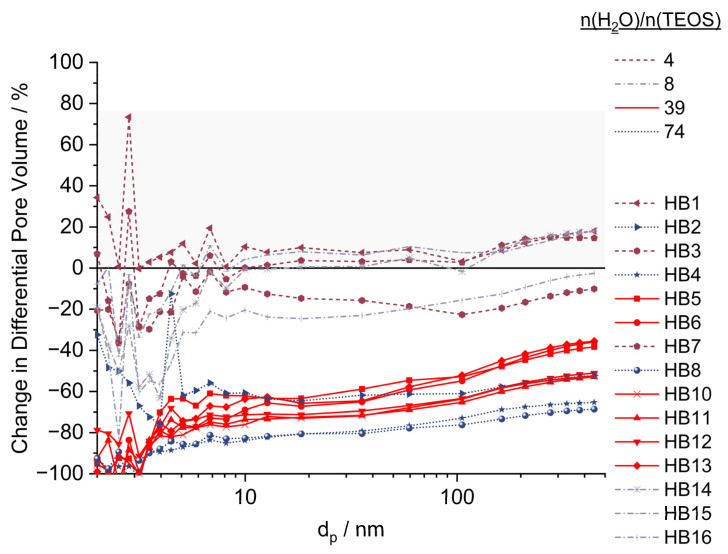
Relative change in pore volume distribution dependent on the pore size compared to the template particle. Line colors and types are grouped according to n(H_2_O)/n(TEOS) level setting: n(H_2_O)/n(TEOS) = 4 as burgundy dashed line, n/n = 8 as grey dashed-dotted line, n/n = 39 as solid red line and n/n = 74 as dotted blue line.

Figure 6 compares the relative changes in pore volume as a function of the pore diameter of the HBs and the template. From these data, it is deduced how the process factor settings influence the deposition of silica as a function of the pore size.

The most prominent difference in the relative V_p_ change compared to the porous template p@TEPA occurs at pore sizes < 5 nm. Samples that exhibit an increase in V_p_ are HB1 (−/−), HB7 (−/0) and, to some extent, HB14 (8/0) and HB15 (8/−). Apart from HB1, which shows an increase in the pore volume across the entire pore size range, the relative pore volume for pore sizes < 10 nm decreases for HB7, HB14 and HB15 as a result of silica deposition in small pores. Starting from pore sizes > 10 nm, the relative pore volume also increases compared to the template. These samples were prepared with low water-to-TEOS ratios and low ammonia concentrations. This group of particles is found in the lower left corner of the experimental design space (Figure 4a). Here, very small mass depositions of SiO_2_ were observed, with values of <1 wt% (except for H14 2.67 wt%). In this region of the experimental design space, the deposition of silica is almost negligible. In contrast, the pores of these HB particles are observed to be enlarged. Lacking protective silica deposits on the pore walls, in these HBs, the pores are still accessible to the solvent. This can lead to mechanical washing-out effects and the dissolution of non-crosslinked small polymer residues into the continuous phase of the sol-gel process, which consists of isopropyl alcohol. This external influence superimposes the effects of the controlled process factor variations. For all other samples, where the silica deposition prevents solubility and mechanical washing-out effects, this external influence on the relative pore volume changes does not play a role. Here, the change in pore volume can be fully explained by the following controlled process factors: water-to-precursor ratio and ammonia concentration.

The decrease in pore volume is most pronounced for small pores, which are simply closed by silica deposition. Syntheses, which were carried out at low ratios of n(H_2_O)/n(TEOS), display the lowest silica deposition and the smallest loss of pore volume at pore diameters of <10 nm. Syntheses with medium (CPs, in red) or high water/precursor ratios lead to an almost total closure of small pores < 5 nm (–80 to –100 %). Above around 5 nm, the decrease is then very uniform, with a slight increase in preserved pore volume for larger pores. This indicates the formation of a uniformly thick silica layer on the pore walls. These findings suggest an adsorptive deposition of silica onto the p@TEPA pore walls.

Although the response variables correlate well with each other linearly (Figure 5), they are each affected by the varied process factors in different ways and strengths. Table 3 shows the relative factor effect strengths, with the factors normalized to factor A–n(H_2_O)/n(TEOS). This allows for a comparison of the relative strengths of the effects for the factor B–c(NH_3_), the synergistic interaction effect AB and the nonlinear effect terms A² and B² between the models for the respective response variable.

The effect of c(NH_3_) always has the same algebraic sign as the effect term of factor A in all models and therefore always acts in the same direction as the ratio n(H_2_O)/n(TEOS). However, the effect of the ammonia concentration is always smaller than the effect of the stoichiometric ratio of water to TEOS, except for the model of the median pore size. The SiO_2_ content and V_p_ not only show a strong negative correlation (Figure 2b,l) but are also very similar in terms of relative effect strengths, but with opposing effect directions. With normalized factor strengths for the ammonia concentration of B = + 0.27 and B = –0.26 and an A² = –0.88 and A² = +0.71 for SiO_2_ content and V_p_, respectively, the V_p_ declines gradually with SNP deposition inside the porous template. The deposition of SNPs thus takes place inside the porous network of the template within the observed experimental space, except for HB4 and HB8, where non-porous secondary particles were formed. Here, a critical factor-level combination of A = 60 and B = 45.6 mmol∙L^−1^ was exceeded.

In contrast to the V_p_, the SSA shows a non-linear dependence on the ammonia concentration. The effect strength of ammonia is nearly twice as strong, but the decline in SSA with an increasing ammonia concentration levels off. This results from the deposition of SNPs at the outer surface of the template, which leads to a rougher surface texture of the HBs and an increase in the available particle surface (Figure 3g and Figure 4b). This counterbalances the steep decline in SSA due to the closure of pores, which prevents the accessibility of the inner pore walls, even for small molecules such as nitrogen.

For all models, the linear A term is coupled to a non-linear A² in an opposing effect direction. This means that the ratio of n(H_2_O)/n(TEOS) always has a non-linear component which leads to the diminishing of the overall influence with increasing water-to-precursor ratios n(H_2_O)/n(TEOS). The non-linear behavior of the water-to-TEOS ratio has already been discussed regarding the size of the SNPs by other groups and was summarized by Bourebab et al. [48], who report a maximum size of the SNPs occurring between 10 and 35 wt% water. In the present study, the maximum size of the SNP was at an approximate water content of 20 wt%, which corresponds to an n(H_2_O)/n(TEOS) ratio of 60. When this critical level was exceeded, a comparatively smaller increase in SNP size with increasing water content was found, which is reflected by the statistically significant A² term. Here, with the factor-level combination used as the starting conditions in the synthesis of HB4 and HB8 (Figure 4b and Figure A3), the formation of secondary particles in the continuous phase was observed. As the hydrolysis and condensation rates increase with the increase in n(H_2_O)/n(TEOS), along with simultaneously elevated levels of ammonia, the fast-growing SNPs become too large to penetrate into the pores of the template.

Since all HB particles which result from the combinations of factor-level settings according to the FCD were prepared at a pH between 10.5 and 11.0, the amino groups of the TEPA functionalization are still partly protonated, and, thus, the template carries a positive surface charge [46]. At the same time, the surface near silanol groups of the SNPs, which are produced in the continuous phase, are deprotonated and form negatively charged –Si-O^−^functions. Small SNPs have a higher surface-to-volume ratio than larger ones and, thus, a higher silanol concentration [4]. This results in a high negative charge-to-volume ratio coupled with an increased mobility for small SNPs [49]. As a consequence of the strong electrostatic attraction of the negatively charged SNPs to the positively charged amino-functionalized polymer surface, SNPs are readily incorporated into the porous network of the p@TEPA template. The p@TEPA template acts like a filter which removes small SNPs permanently from the reaction medium, withdraws them from reaction equilibria and thus prevents further growth. Therefore, as long as the rate of the diffusion of SNPs into the porous template exceeds the rate of particle growth, they cannot grow beyond a certain size on the outside, despite a sufficient monomer being available. This is the case at low n(H_2_O)/n(TEOS) ratios. On the other hand, monomers and other hydrolyzed silanol species do not diffuse into the template as they rapidly undergo hydrolysis and condensation reactions in the continuous phase. Inside the porous network of the template, the small silica particles adsorb onto the positively charged pore walls, agglomerate and form uniform layers on the pore walls.

**Figure 7:**
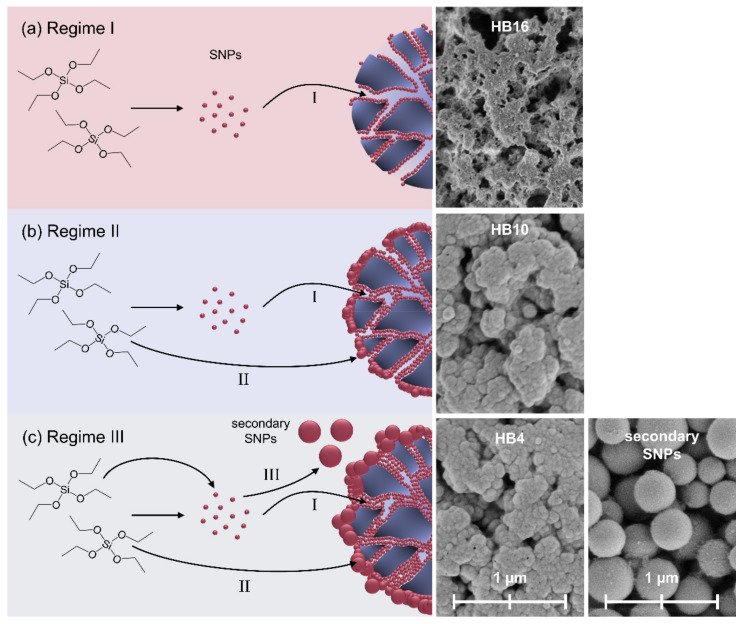
(**a**) Regime I corresponds to relatively low water values, (**b**) Regime II corresponds to medium values and (**c**) Regime III corresponds to high water-to-precursor ratios. Exemplarily, the regions are derived from the particles HB16, HB10 and HB4, respectively. It shows the diffusion of SNPs, after exceeding the supersaturation concentration, into the template. There, they aggregate in the absence of monomer (I). The hydrolysis is accelerated (Regime II), which deposits in the pores (I) and on the HB surface, where they undergo both the aggregation of SNPs and monomer addition, as they are in contact with the reaction medium (II). In Regime III, with highest hydrolysis/condensation rate, SNPs additionally undergo monomer addition (III) in the continuous phase, forming secondary particles, which are not attached to the HB.

By the systematic variation in the critical process factors within our experimental design space, HBs with different morphological characteristics were produced, the formation of which can be assigned to three reaction regimes depending on the sol-gel starting conditions (Figure 7).

At low water-to-TEOS ratios, hydrolysis is slow. The small SNPs formed are readily attracted by the template and are completely deposited inside the pores of p@TEPA. Thus, few silica particles are formed that attach to the pore walls on the inside of the template (HB1, 3, 7, 14–16) (Regime I, Figure 7b). If the water ratio is increased (HBs 2, 9, 10 and CPs) and the rate of hydrolysis is accelerated [28], the amount of silica deposition is increased. Those particles that are deposited on the outer surface of the template are still in contact with the reaction medium and continue to grow by monomer addition (Region II, Figure 7c).

The particle growth is further promoted by increasing the ammonia concentration [30]. This behavior affects the size of the SNP on the outer surface of the HBs and the HB pore size median (Figure 2d–f,n–p). Furthermore, more SNPs are deposited in the pores on the inside of the HBs, resulting in thicker silica layers covering the pore walls.

If hydrolysis and condensation rates are increased to an extent at which they exceed the diffusion of the SNPs into the p@TEPA pores, the SNPs remain in the reaction medium. There, they undergo further growth and form nonporous secondary particles (Region III Figure 7c). This is the case for n(H_2_O)/n(TEOS) ≥ 60 combined with a c(NH_3_) of 45.6 mmol∙L^−1^ or higher (HB4 and 8; see Appendix B Figure A3).

## 4. Materials and Methods

### 4.1. Chemicals

Ammonia solution (28–30%), benzoyl peroxide (BPO, 75%), dibutyl phthalate (DBP, 99%), glycidyl methacrylate (GMA ≥ 97%), tetraethyl orthosilicate (TEOS) and toluene (anhydrous 99.8%) were purchased from Sigma-Aldrich Chemie GmbH (Traufkirchen Germany). Polyvinyl alcohol (PVA, hydrolyzed 86–89%) was supplied from abcr GmbH (Karlsruhe, Germany). Cyclohexanol (99%), ethylene glycole dimethacrylate (EDMA, 98%), HPLC grade water and tetraethylene pentamine (TEPA) were purchased from Fisher Scientific GmbH (Schwerte, Germany). Sodium dodecyl sulfate (SDS ≥ 99%) and 2-propanol (HPLC grade > 99.8 %) were purchased from Carl Roth GmbH + Co. KG (Karlsruhe, Germany). All chemicals were used as received.

### 4.2. Preparation of HBs

The hybrid microspheres were synthesized using a polymeric hard template. The porous *p*(GMA-*co*-EDMA) particles were synthesized according to the procedure described previously [17], with a scale-up. Further information is given in Appendix A.

The HBs were synthesized under basic hydrolysis-condensation conditions. The ammonia concentration and the molar ratio of n(H_2_O)/n(TEOS) were systematically changed according to a face-centered-central composite design (FCD) to assess the synthesis parameter effects on HB particle morphology, the surface and pore characteristics as well as the silica deposition behavior. The range of process factor levels is given in Table 4.

The absolute amount of TEOS was the same for all particle syntheses. Depending on the molar ratio of n(H_2_O)/n(TEOS), the amount of additional water was calculated and varied accordingly. The water content of the NH_4_OH solution was included in these calculations. A total volume of 140 mL was used, and the volume of the reaction mixtures was adjusted using 2-propanol. The changes in 2-propanol volume were <8%. Generally, 2 g of the p@TEPA particles were dispersed in a 2-propanol/H_2_O mixture and sonicated for 5 min. Then, the NH_4_OH solution was added as the basic catalyst under stirring, followed by the addition of 21.5 mmol TEOS. The mixture was stirred with 550 rpm for 24 h at room temperature. The HBs were filtered off and washed three times with ethanol and water. The particles were then dried at room temperature prior to analysis.

### 4.3. Nitrogen Adsorption Measurements

For the determination of the specific surface area, pore size and pore volume, the BELSORP MiniX (Microtrac Retsch GmbH, Haan, Germany) was used for nitrogen adsorption measurements. The sample mass was ~ 150 mg (145 ± 14 mg), except for samples with a very small surface area, which were measured with an increased sample amount of ~300 mg (HB4 with 291 mg and HB8 with 290 mg). All samples were degassed at 30 °C for 24 h up to a final vacuum of about 2∙10^−2^ mbar to eliminate possible physisorbed substances and to achieve a reproducible intermediate state [50]. The samples were evacuated using the BELSORP VACII (Microtrac Retsch GmbH, Haan, Germany). The N_2_-adsorption and desorption measurements were performed at 77 K. The analysis of adsorption and desorption isotherms was performed using the BELMaster 7 software (7.3.2.0, Microtrac Retsch GmbH, Haan, Germany). The specific surface area was analyzed according to the Brunauer–Emmet–Teller method (BET) [51,52]. For this, the relative pressure range of *p/p*_0_ 0.05–0.3 was used [47]. The pore size distribution was determined using the Barrett–Joyner–Halenda (BJH) method from the desorption branch and the Harkins–Jura standard isotherm. The pore size diameters were analyzed in the range from 2 to 500 nm. The pore volume was determined from a single adsorption point at *p/p*_0_ of 0.95.

### 4.4. Scanning Electron Microscopy Images (SEM)

SEM images were acquired using a Hitachi SU8030 (Hitachi High-Tech Europe GmbH, Krefeld, Germany) to assess surface morphology, particle size and dispersity. A self-written MATLAB script was used to semi-automatically measure the size and dispersity from the SEM images. At least 120 particles were measured. The dispersity is given by the d_90_/d_10_ value which indicates the width of the particle size distribution. Here, d_90_ is the value below which 90% of the measured values of the particle sizes lie. d_10_ is the value below which 10% of the measured particle sizes lie. A d_90_/d_10_ value smaller than 1.4 was considered as a monodisperse distribution.

### 4.5. Thermogravimetric (TGA) Determination of SiO_2_ Content

The amount of deposited SiO_2_ content was determined using thermogravimetric analysis (TGA) using the TGA/DSC I from Mettler Toledo. Portions of a 119.9–121.4 mg (125.8 ± 3.1 mg) sample were weighed into a 900 µL alumina crucible, and thermal analysis was performed under synthetic air (50 mL/min). A heating rate of 5 K/min in a temperature range from 30 to 800 °C was performed. The relative mass of the remaining residue is the proportion of SiO_2_ to the mass of the hybrid material.

### 4.6. Experimental Design

The mathematical description of linear, non-linear and interaction effect terms [36,53] was carried out by systematic analysis based on an FCD. Then, 16 HB particle batches were synthesized with five center point (CP) replications to determine reproducibility and system variance. Three additional syntheses were conducted with n(H_2_O)/n(TEOS) = 8 to support model stability in the critical model region with low molar ratios. The effects of two factors and their possible synergistic interactions, c(NH_3_) and n(H_2_O)/n(TEOS), were investigated by systematic variations in the factor-level settings according to the FCD response surface design. The factor levels for low, intermediate and high settings are given in Table 4. The model effect terms were analyzed using analysis of variance (ANOVA). A model or model term was considered statistically significant when its *p*-value was *p* ≤ 0.05. For model conceptualization and analysis, the Design Expert DX12 (Version 12.0.12.0, Stat-Ease Inc., Minneapolis, MN, USA) software package was used.

## 5. Conclusions

The process factors n(H_2_O)/n(TEOS) and c(NH_3_) specifically control the silica deposition on the inside and the outside of the p@TEPA template. Three discrete process regimes were identified. At low water conditions, only pores < 5 nm are filled with deposited silica to some extent. At medium to high water-to-precursor ratios, pores < 5 nm are almost completely filled with silica, and the pore volume of pores > 5 nm decreases uniformly. This suggests the formation of a uniform silica layer inside the porous template network through primary particle adsorption at the pore walls and aggregation within the porous network of the template. The present work demonstrates under which conditions the pores are closed and in which way and presents a mechanistic proposal for the construction of a silica network in a porous organic template.

This allows for access to new tailor-made hybrid materials with different properties. Specific hybrid materials can be created in which the organic template is coated with a thin silica layer, e.g., to protect the organic matter. These silica layers covering the pore walls can be further built up until the pores are filled with silica.

Furthermore, by extending the presented experimental space towards even higher ammonia and water concentrations and thus higher rates of hydrolysis and condensation, the formation of large SNPs exceeds the rate of diffusion, which hinders the incorporation of SNPs into the porous network of the template. This leads to the formation of particles with a porous organic core protected by a silica shell.

## Figures and Tables

**Table 1:** Factor-level settings and their corresponding particle properties particle size, dispersity, specific surface area, pore diameter, pore volume (at *p/p*_0_ = 0.95), SiO_2_ content and size of the SNPs. The experiments are listed according to Yates standard order (Std). * Outlier-HB9 is marked in italics and was not included in the model building. ^†^ No SNPs visible or not measurable (below image resolution of SEM).

	Factor-Level Settings		Response Values						
	A n(H_2_O)/ n(TEOS)	B n(NH_3_)	Particle Size	d_90_/d_10_	SSA	Pore Diameter Φ_50_	Pore Volume V_p_	SiO_2_ Content	SNP Size
		/mmol∙L^−1^	/µm		/m²∙g^−1^	/nm	/cm^3^∙g^−1^	/wt%	/nm
**p@TEPA**	-	-	8.3	1.04	63.79	13.0	0.12	0	-
**HB1**	4	17.1	8.3	1.11	73.87	12.9	0.13	0.1	0 ^†^
**HB2**	74	17.1	8.6	1.14	35.11	12.6	0.03	19.7	20
**HB3**	4	74.1	8.6	1.06	55.86	12.7	0.10	2.7	0
**HB4**	74	74.1	8.6	1.07	8.75	18.1	0.02	31.2	66
**HB5**	39	45.6	8.5	1.09	20.87	16.1	0.04	22.6	27
**HB6**	39	45.6	8.5	1.08	12.92	17.8	0.03	26.9	35
**HB7**	4	45.6	8.5	1.06	62.72	13.8	0.12	0.4	0 ^†^
**HB8**	74	45.6	8.6	1.06	9.63	16.5	0.02	30.1	47
HB9 *	*39*	*17.1*	*8.6*	*1.05*	*47.38*	*17.5*	* 0.09	* 13.0	*24*
**HB10**	39	74.1	8.7	1.07	12.02	17.1	0.03	28.6	58
**HB11**	39	45.6	8.6	1.06	13.51	16.3	0.03	26.7	35
**HB12**	39	45.6	8.5	1.07	17.10	15.5	0.05	25.9	43
**HB13**	39	45.6	8.5	1.08	14.32	17.0	0.03	25.9	40
**HB14**	8	45.6	8.5	1.07	50.76	15.5	0.10	2.7	0 ^†^
**HB15**	8	17.1	8.5	1.06	64.03	14.2	0.12	0.2	0 ^†^
**HB16**	8	74.1	8.3	1.15	47.03	14.9	0.09	7.9	18

**Table 2:** Excerpt from the analysis of variance (ANOVA) tables with *p*-values for the response surface models of the target response variables particle size, SiO_2_ content, median pore diameter (Φ_50_), specific surface area (SSA), pore volume (V_p_) and size of the SNPs, as well as their fit statistics. Complete ANOVA tables for each model are given in Appendix C Table A1, Table A2, Table A3, Table A4, Table A5 and Table A6.

*p*-Values						
Response	Particle Size	SiO_2_	Φ_50_	SSA	V_p_ (*p/p*_0_ = 0.95)	SNP Size
	/µm	/wt%	/nm	/m²∙g^−1^	/cm³∙g^−1^	/nm
**Model**	0.0172	<0.0001	0.0007	<0.0001	<0.0001	<0.0001
**A**–n(H_2_O)/n(TEOS)	0.0172	<0.0001	0.0099	<0.0001	0.0007	<0.0001
**B**–c(NH_3_)	n.s.	0.0011	0.0051	<0.0001	<0.0001	<0.0001
**AB**	n.s.	n.s.	0.0074	n.s.	n.s.	0.0026
**A²**	n.s.	<0.0001	0.0050	<0.0001	<0.0001	0.0009
**B²**	n.s.	n.s.	n.s.	0.0146	n.s.	n.s.
**Lack of fit**	0.0163	0.2204 (n.s.)	0.4990 (n.s.)	0.4397 (n.s.)	0.9670 (n.s.)	0.6132 (n.s.)
**R²**	0.3624	0.9780	0.8299	0.9840	0.9800	0.9545
**R²_adj_**	0.3152	0.9720	0.7618	0.9776	0.9745	0.9364
**R²_pred_**	0.1684	0.9459	0.5529	0.9535	0.9635	0.8874

n.s. = not statistically significant.

**Table 3:** Relative effect strength normalized to A = ±1.00 in terms of coded factors for particle size, SiO_2_ content, median pore diameter, specific surface area, pore volume and SNP size.

	SiO_2_	Φ_50_	SSA	V_p_	SNP Size
	/wt%	/nm	/m²∙g^−1^	/cm³∙g^−1^	/nm
**A**–n(H_2_O)/n(TEOS)	+1.00	+1.00	−1.00	−1.00	+1.00
**B**–c(NH_3_),	+0.27	+1.25	−0.45	−0.26	+0.66
**AB**		+1.30			+0.45
**A²**	−0.88	−1.78	+0.92	+0.71	−0.68
**B²**			+0.28		

**Table 4:** Range of process factor-level settings as used in the face-centered-central composite experimental design (FCD).

Factor	Name	Low Setting (−)	Center Point (0)	High Setting (+)
A	n(H_2_O)/n(TEOS)	4	39	74
B	c(NH_3_)/mmol	2.39	6.38	10.37

## Data Availability

The data presented in this study are available within this article or are available from the authors upon request.

## Appendix A. Preparation of Amino-Functionalized Porous Polymer Template

First, 0.9 g monodisperse polystyrene seeds (1.95 µm, of d_90_/d_10_ = 1.09) were sonicated for 10 min in 15 mL of a 2.0 g∙L^−1^ SDS solution. An emulsion of 6.0 mL DBP and 450 mL of a 2.53 g∙L^−1^ SDS solution (10 min at 5000 rpm) were added to the seed particle suspension. The suspension was stirred for 24 h at 200 rpm at room temperature. Then, 450 mL of SDS (3.33 g∙L^−1^), the initiator BPO (1.2 g), 22.5 mL of each cyclohexanol, EDMA, GMA and toluene were homogenized for 10 min at 5000 rpm. The emulsion was added, and the system was stirred at 200 rpm for another 24 h. Then, 450 mL of a PVA solution (23.3 g∙L^−1^) was added to the mixture as a stabilizer. The reaction mixture was stirred at 200 rpm for 24 h at 70 °C for polymerization. Thereafter, the porous *p*(GMA-*co*-EDMA) particles were washed three times with ethanol and three times with water.

For amino functionalization, 35 g of dried *p*(GMA-*co*-EDMA) particles were suspended in 1400 mL of deionized water and sonicated for 15 min. Then, 0.256 mol of TEPA were added dropwise under stirring (200 rpm). After the addition, the mixture was heated to 80 °C for 24 h. The functionalized particles were filtered off and washed three times with ethanol and water. The particles will be further referred to as p@TEPA.

## Appendix C. ANOVA Tables for the RSMs

**Table A1:** Analysis of variance (ANOVA) for the analysis of the FCD design of the median particle size.

Source	Sum of Squares	df	Mean Square	F-Value	*p*-Value	
Model	0.0561	1	0.0561	7.45	0.0172	significant
A-n(H_2_O)/n(TEOS)	0.0561	1	0.0561	7.45	0.0172	
Residual	0.0980	13	0.0075			
Lack of Fit	0.0943	9	0.0105	11.26	0.0163	significant
Pure Error	0.0037	4	0.0009			
Cor Total	0.1541	14				

**Table A2:** Analysis of variance (ANOVA) for the analysis of the FCD design of SiO_2_ content.

Source	Sum of Squares	df	Mean Square	F-Value	*p*-Value	
Model	2187.31	3	729.10	162.91	<0.0001	significant
A-n(H_2_O)/n(TEOS)	1381.35	1	1381.35	308.65	<0.0001	
B-c(NH3)	86.00	1	86.00	19.21	0.0011	
A²	432.20	1	432.20	96.57	<0.0001	
Residual	49.23	11	4.48			
Lack of Fit	39.41	7	5.63	2.29	0.2204	not significant
Pure Error	9.82	4	2.45			
Cor Total	2236.54	14				

**Table A3:** Analysis of variance (ANOVA) for the analysis of the FCD design of the median pore size ϕ_50_.

Source	Sum of Squares	df	Mean Square	F-Value	*p*-Value	
Model	38.43	4	9.61	12.20	0.0007	significant
A-n(H_2_O)/n(TEOS)	7.93	1	7.93	10.07	0.0099	
B-c(NH3)	10.03	1	10.03	12.74	0.0051	
AB	8.81	1	8.81	11.19	0.0074	
A²	10.08	1	10.08	12.79	0.0050	
Residual	7.88	10	0.7879			
Lack of Fit	4.84	6	0.8074	1.06	0.4990	not significant
Pure Error	3.03	4	0.7586			
Cor Total	46.31	14				

**Table A4:** Analysis of variance (ANOVA) for the analysis of the FCD design of the SSA.

Source	Sum of Squares	df	Mean Square	F-Value	*p*-Value	
Model	7539.12	4	1884.78	153.80	<0.0001	significant
A-n(H_2_O)/n(TEOS)	3882.36	1	3882.36	316.80	<0.0001	
B-c(NH3)	681.83	1	681.83	55.64	<0.0001	
A²	989.61	1	989.61	80.75	<0.0001	
B²	106.41	1	106.41	8.68	0.0146	
Residual	122.55	10	12.25			
Lack of Fit	79.47	6	13.25	1.23	0.4397	not significant
Pure Error	43.08	4	10.77			
Cor Total	7661.67	14				

**Table A5:** Analysis of variance (ANOVA) for the analysis of the FCD design of the V_p_.

Source	Sum of Squares	df	Mean Square	F-Value	*p*-Value	
Model	0.0249	3	0.0083	179.66	<0.0001	significant
A-n(H_2_O)/n(TEOS)	0.0174	1	0.0174	375.80	<0.0001	
B-c(NH3)	0.0010	1	0.0010	21.98	0.0007	
A²	0.0036	1	0.0036	76.88	<0.0001	
Residual	0.0005	11	0.0000			
Lack of Fit	0.0001	7	0.0000	0.2028	0.9670	not significant
Pure Error	0.0004	4	0.0001			
Cor Total	0.0254	14				

**Table A6:** Analysis of variance (ANOVA) for the analysis of the FCD design of the SNP size.

Source	Sum of Squares	df	Mean Square	F-Value	*p*-Value	
Model	6865.92	4	1716.48	52.49	<0.0001	significant
A-n(H2O)/n(TEOS)	3797.04	1	3797.04	116.11	<0.0001	
B-c(NH3)	1346.92	1	1346.92	41.19	<0.0001	
AB	517.00	1	517.00	15.81	0.0026	
A²	699.79	1	699.79	21.40	0.0009	
Residual	327.01	10	32.70			
Lack of Fit	179.01	6	29.84	0.8064	0.6132	not significant
Pure Error	148.00	4	37.00			
Cor Total	7192.93	14				
